# Piloting a brief assessment to capture consumption of whole plant food and water: version 1.0 of the American College of Lifestyle Medicine Diet Screener (ACLM Diet Screener)

**DOI:** 10.3389/fnut.2024.1356676

**Published:** 2024-04-26

**Authors:** Micaela C. Karlsen, Kara L. Staffier, Kathryn J. Pollard, Kelly C. Cara, Sarah M. Hulit, Erin K. Campbell, Susan M. Friedman

**Affiliations:** ^1^Department of Research, American College of Lifestyle Medicine, Chesterfield, MO, United States; ^2^Applied Nutrition and Global Public Health, University of New England, Biddeford, ME, United States; ^3^Division of Nutrition Epidemiology and Data Science, Friedman School of Nutrition Science and Policy, Tufts University, Boston, MA, United States; ^4^Department of Public Health Sciences, University of Rochester School of Medicine and Dentistry, Rochester, NY, United States; ^5^Department of Medicine, University of Rochester School of Medicine and Dentistry, Rochester, NY, United States; ^6^Rochester Lifestyle Medicine Institute, Rochester, NY, United States

**Keywords:** nutrition, diet, nutrition assessment, dietary screener, food frequency questionnaire, whole-food plant-based diet, FFQ, WFPB

## Abstract

**Background:**

Despite the availability of various dietary assessment tools, there is a need for a tool aligned with the needs of lifestyle medicine (LM) physicians. Such a tool would be brief, aimed at use in a clinical setting, and focused on a “food as medicine” approach consistent with recommendations for a diet based predominately on whole plant foods. The objective of this study is to describe the development and initial pilot testing of a brief, dietary screener to assess the proportion of whole, unrefined plant foods and water relative to total food and beverage intake.

**Methods:**

A multidisciplinary study team led the screener development, providing input on the design and food/beverage items included, and existing published dietary assessment tools were reviewed for relevance. Feedback was solicited from LM practitioners in the form of a cross-sectional survey that captured information on medical practice, barriers, and needs in assessing patients’ diets, in addition to an opportunity to complete the screener and provide feedback on its utility. The study team assessed feedback and revised the screener accordingly, which included seeking and incorporating feedback on additional food items to be included from subject matter experts in specific cultural and ethnic groups in the United States. The final screener was submitted for professional design, and scoring was developed.

**Results:**

Of 539 total participants, the majority reported assessing diet either informally (62%) or formally (26%) during patient encounters, and 73% reported discussing diet with all or most of their patients. Participants also reported facing barriers (80%) to assessing diet. Eighty-eight percent believed the screener was quick enough to use in a clinical setting, and 68% reported they would use it.

**Conclusion:**

The ACLM Diet Screener was developed through iterative review and pilot testing. The screener is a brief, 27-item diet assessment tool that can be successfully used in clinical settings to track patient dietary intakes, guide clinical conversations, and support nutrition prescriptions. Pilot testing of the screener found strong alignment with clinician needs for assessing a patient’s intake of whole plant food and water relative to the overall diet. Future research will involve pilot testing the screener in clinical interventions and conducting a validation study to establish construct validity.

## Introduction

Health professionals have a need for brief dietary assessment tools that align with the recommendations they give patients. There is increased interest in implementing “food as medicine” or “food is medicine” and bridging what is known in nutrition research to clinical settings. This aligns with the approach to care practiced in the field of lifestyle medicine (LM), in which dietitians, health coaches, and other members of the interdisciplinary team work closely with physicians to support a coordinated care approach that incorporates the use of nutrition prescriptions (1).

Lifestyle medicine clinicians typically recommend a diet based predominantly on whole, plant foods and water as the beverage of choice. Increasingly, research finds that plant-based diets of all kinds are associated with lower risk for chronic disease (2), and are effective interventions for healthy weight loss (3), improvement of blood glucose control (4, 5), treatment of cardiovascular disease (6, 7), and even remission of type 2 diabetes (8, 9). In addition, plant-based diets can range in definition from vegan and vegetarian, to flexitarian, and even include other omnivorous diets such as Mediterranean and DASH that incorporate more unrefined plant foods than a typical Western diet (10). To support treatment protocols and behavior change in patients, LM clinicians need a questionnaire that has a low respondent burden, is easy and quick to implement in the context of brief patient encounters, and provides simple and straightforward feedback on the amount of whole plant food and water being consumed relative to the overall diet as captured by the screener (referred to from here on as “overall diet”). Additionally, the ideal questionnaire would provide sufficient information to thoroughly capture not only intake of whole foods, but also consumption of processed food and ultra-processed food, as nearly 60% of total energy intake in the United States comes from ultra-processed foods (11). Responses on such a questionnaire, or screener, would provide a clear path for health professionals to guide clinical conversations around dietary improvements with patients.

Dietary screeners, sometimes called scanners, are short dietary assessment tools designed to capture self-reported intake of particular aspects of an individual’s diet in a quick and easy-to-use questionnaire format. Similar to a food frequency questionnaire (FFQ), screeners typically focus only on frequency of consumption (12) and ask about usual intake patterns over a period of time (e.g., the past 30 days or the past year) rather than exact quantities, portions, or brands consumed on a given day (13). Unlike FFQs, which are commonly designed to assess the whole diet by asking about multiple food groups and seasonal changes in intake, screeners often focus on a single nutrient (e.g., NutritionQuest’s Fat Intake Screener) (14), food (e.g., Block Soy Foods Screener) (14, 15), or food component (e.g., Beverage Questionnaire) (16), or select food groups (e.g., NutritionQuest’s Fruit, Vegetable, and Fiber Screener) (17). However, screeners have also been developed to capture several components across the diet [e.g., the Dietary Screener Questionnaire (18, 19) which captures fruit, vegetable, dairy/calcium, added sugars, whole/grains/fiber, red meat, and processed meat; or the Nova Ultra-processed Food Screener (20)]. There are a multitude of dietary assessment tools available, ranging from food records, 24 h recalls and FFQs to brief screeners of fewer questions, such as those from the Register of Validated Short Dietary Assessment Instruments (RSVSDAI) at the National Cancer Institute (NCI) (21). Each tool focuses on capturing a different aspect of the diet, therefore enabling different specific comparisons or analyses. Brief screener tools are often most appropriate for clinical settings in which diet is discussed, where there is limited time available to conduct assessments (22).

Due to their focus, simplicity, and shorter length, screeners capture less detailed information than dietary assessment tools such as 24-h recalls and food records. Despite this, their simplicity means they can be used to capture a general sense of an individual’s or population’s intake of dietary components of specific interest both quickly (in under 15 min) (23) and inexpensively when a great level of detail is neither necessary nor feasible. Screeners can be used to track changes in usual intake over time and to compare high and low levels of intake within a population (24). Where more detail or additional information is needed, screeners can be combined with other tools.

Each tool focuses on capturing a different aspect of the diet, therefore enabling different specific comparisons or analyses (21). Within the RSVSDAI at NCI (21), a validated brief dietary assessment that is a low-tech, simple questionnaire measuring whole plant food and water consumption does not exist. To fill this gap, this study team undertook the development of a new dietary assessment tool, the ACLM Diet Screener. The objective of this study is to describe the development and initial pilot testing of the screener, in preparation for future validation of this tool.

## Methods

The screener development followed an iterative process beginning in September of 2021. A study team was assembled that included lifestyle medicine physicians, nutrition researchers, and staff from the sponsoring organization. The study team met weekly through August 2023, initially with the goal of developing a longer dietary intake questionnaire for use in research settings. However, discussion emerged about the need for a brief dietary assessment tool that could be used during patient encounters in clinical settings. Subsequently, the study team decided to create two tools: a longer dietary intake questionnaire for research use and a brief diet screener suitable for clinical encounters of limited duration. Initial development and pilot testing of the brief diet screener is presented here.

The following resources relevant to plant-based dietary patterns were reviewed to identify gaps in food items recorded or analyzed by existing assessments or scores, as well as to identify potential foods and beverages for inclusion on the screener: (1) a theoretical analysis of a whole food, plant-based dietary pattern (25), (2) United States Dietary Guidelines and MyPlate (26), (3) the Vegetarian Food Guide Pyramid (27), (4) the Healthy Eating Index (28), (5) the Alternative Healthy Eating Index (29, 30), (6) the DASH diet (31, 32), and (7) the Mediterranean Diet Score (33). Informed by these existing scores and resources, as well as food categories consistent with the ACLM dietary position statement recommending an eating plan based predominantly on a variety of minimally processed vegetables, fruits, whole grains, legumes, nuts, and seeds (34), the study team discussed food items to include and how to group them into categories most useful for LM clinicians, as well as possible categories representing frequency of consumption. A goal was set to differentiate between infrequent consumption and zero consumption of a food, to enable LM clinicians to continue more nuanced conversations with patients about dietary improvements.

An initial draft of the screener was created and informally shared with *N* = 81 individuals (friends, family, and colleagues of the study team). Based on feedback that emerged, it was determined that food examples would be useful in understanding how to answer the screener. The team continued weekly meetings and developed food examples to provide context for how participants should interpret each food category when responding.

Finally, sets of additional questions on the following factors and dietary behaviors were included, based on relevance to long-term behavior change interventions: food security, supplement intake, food preferences, eating style, food preparation behaviors, and self-efficacy around healthy food intake. These questions are intended to be used as optional modules in situations where the healthcare practitioner would find the information useful for discussion during a clinical visit. The two-question food security screener was also included (35).

Two academic, senior scientists with expertise in nutritional epidemiology and dietary assessment methods, as well as a senior statistician with experience in dietary assessment were engaged as consultants to provide feedback on the structure of the screener questions and answer choices, taking into consideration the long-term goal of validation.

The screener, originally containing 25 items and asking about intake over the previous 4 weeks, was pilot-tested with an online survey administered to lifestyle medicine clinicians, with data collection from February 20, 2023 to April 18, 2023. This study was reviewed by the University of New England IRB.

Participants were recruited by the sponsoring organization through email, social media posts, and resharing online by individuals. Recruitment targeted healthcare practitioners, but non-practitioners were not specifically excluded and also participated. Individuals reviewed a participant information sheet and indicated that they were 18 years of age or older and consented to participate by beginning the survey. The survey captured demographics and medical practice status, degree and specialty, barriers and needs in assessing patients’ diets, and characteristics of the practitioner’s patient population. Participants then completed the screener itself and answered both multiple choice (Likert scales) and free text questions on the utility of the screener, their likelihood of using it, overall feedback on the screener, and possible formats summarizing intake reported on the screener. Time required to take the screener was assessed with start time and end time questions immediately before and after the dietary screener, as it was embedded in the larger survey. Finally, participants answered sets of optional questions on dietary behaviors and provided feedback about these questions using a Likert scale of how likely they were to use these optional questions. Participants were provided the opportunity to give unstructured, free-text feedback on any aspect of the screener or other questions.

Quantitative data were descriptively analyzed using SAS software 9.4 (Cary, NC, United States). Survey responses were included in this analysis if respondents answered questions on start time, end time, and the last multiple-choice question asking for screener feedback on whether they preferred a pie chart or bar chart for presenting the results. Time required to take the screener was calculated after dropping *n* = 21 outlier observations that appeared to be due to leaving the browser open for extended periods of time (>20 min). Screener feedback in the form of free-text data was coded into categories of responses by at least two members of the research team, with a third team member participating in discussions to resolve conflicts. Initially, the free-text data were evaluated by the entire research team in several of the weekly meetings to calibrate the coding. Responses that were difficult to code were brought to the team for evaluation throughout the coding process.

Once all the feedback about the screener was coded, the PI (MK) summarized the findings and first presented the research team with themes of feedback that were straightforward and easy to address (e.g., change wording in the screener, add examples of food items). Second, the PI highlighted themes of feedback that were less straightforward and merited group discussion, and these themes were discussed until consensus was reached around whether and how to revise the screener accordingly (i.e., adding new food categories or changing what was included in them). Finally, the group reviewed feedback that was deemed out of the scope of the project to verify consensus.

Once this initial phase of testing and review was completed, a second phase was initiated to expand and deepen the relevance for the following specific ethnic or cultural groups: African American, Hispanic/Latino, Asian American, Native American, and Indian American. This second phase was designed to maximize alignment with ACLM’s values of diversity and inclusion, to make the screener relevant for as broad an audience as possible, and to respond to the feedback given by pilot testers asking for more culturally relevant and diverse food examples in the screener.

A three-step process was used to adapt the list of food examples. First, ChatGPT (December, 2023) was used for brainstorming to generate food examples for each of the categories with the following query:

*Help me design a dietary screener. Create a list of commonly eaten foods from [XXX] culture that can be used in a nutrition screening questionnaire. Include 5–10 commonly eaten foods in each of the following categories: [insert food categories from screener].*

The query was run six times, replacing “XXX” above with “multiple ethnicities and cultures,” “African American,” “Hispanic Latino,” “Asian American,” “Native American,” and “Indian American.”

Second, between 2 and 5 subject matter experts (SMEs) were recruited for each of the specific groups and asked to review the list of food examples in the diet screener and propose additions or deletions based on their perspective and experience of that culture, with the ChatGPT suggestions provided as a brainstorming aid. Most SMEs were physicians or public health professionals, and all SMEs were members of their relevant demographic group, with the exception of one of the two SMEs reviewing the list of Native American foods, who self-describes as a non-Native public health nutrition researcher who has worked extensively with indigenous communities for over 30 years. Suggestions from SMEs for specific foods were incorporated into the list of example foods, but with a goal of only naming foods or ingredients; specific names of dishes were generally avoided for length reasons.

Third, previously published work on validated dietary screeners or FFQs developed for these specific populations were reviewed. When available, the actual questionnaire or food items were compared to the food examples list in the drafted ACLM Diet Screener. The following list of methods papers and questionnaires were consulted: the Block FFQ adapted for a Hispanic audience (36), a regional FFQ adapted for use among white and African-American adults in the southern United States based on the National Cancer Institute’s Health Habits and History Questionnaire (37), an FFQ developed for African-American women in the midwestern United States (38), the Dietary Screener Questionnaire adapted for an Asian American audience (39), a dietary screener adapted for a Chinese American audience (39), a dietary questionnaire adapted for use in the India Health Study (40), a food frequency questionnaire developed among Canadian First Nations in north-western Ontario (41), an FFQ developed for the Navajo Nation (42), an FFQ developed to evaluate a nutrition intervention for the Apache in Arizona (43), and the OPREVENT2 FFQ that was an expansion of the Block FFQ (44), developed to be used for southwestern and midwestern Native American peoples. In addition, the Block 2014 FFQ (45, 46) and the Harvard semiquantitative FFQ Grid 2007 were reviewed (47, 48).

Food examples from the existing tools that fit into the ACLM Diet Screener categories and were not already included were added.

**Figure 1:**
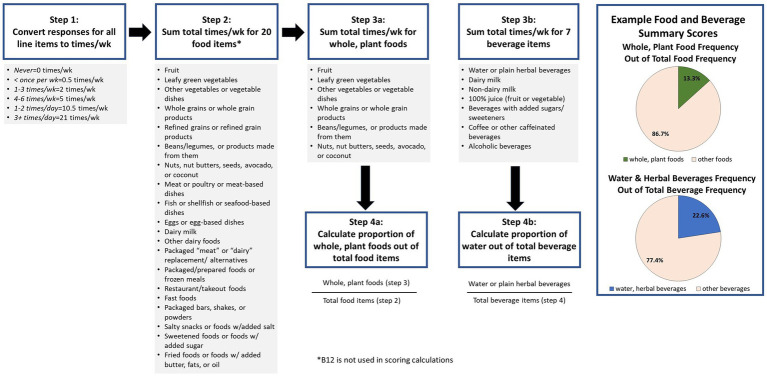
Scoring the ACLM Diet Screener.

A scoring procedure was developed through discussion by the PI and several members of the research team. The scoring (Figure 1) was based on summary scores for frequency of whole plant foods consumed out of total foods reported, and water consumed out of total beverages reported. The final version of the screener including example food lists and the scoring procedure were approved by the study team. Following approval by the team, the screener was then submitted to a professional designer to create a colorized PDF version with photos depicting the food examples as well as a simpler, printer-friendly, black and white PDF version. For ease of use in online settings, images of the food examples were made available.

## Results

The pilot test survey was viewed 4,651 times with *n* = 1,330 total responses (both complete and incomplete). A total of *n* = 505 completed the entire survey, with *n* = 539 answering the critical feedback question and being included in the main analysis, and *n* = 518 being included in the analysis to calculate mean time required to take the screener.

Mean age of participants was 49  years (SD = 12, range 19–80). Reported working status was as follows: 86% answered yes to being in active practice, 6% answered no, 4% reported being in training, and 1% reported being retired/not working, and 2% preferred not to answer. Participants reported practicing in all 50 states, with the most frequently reported states being California (8.0%), New York (5.9%), and Florida (5.2%). Twenty-one percent of the sample reported practicing outside of the United States. Fifty-one percent of respondents were physicians (MD/DO), 15% advance practice providers (NP, PA, APN, and DNP), 15% other clinical/patient care fields (chiropractic, physical therapy, dietitian/nutritionist, and occupational therapy), and 7% nursing (RN, LPN). Physicians reported being boarded in over 20 different medical specialties (American Board of Medical Specialties), with family medicine (31.9%), internal medicine (13.5%), and preventive medicine (7.8%) being the top reported.

Table 1 presents characteristics of practitioners, patients, and the practice. A total of 77% of participants identified as female and 74% as white. A total of 81, 41, and 26% of participants reported serving patient populations with substantial numbers (at least 20%) of white, Black or African-American, and Asian patients, respectively. Participants also reported substantial numbers of patients facing a variety of risk factors for health disparities, with the top three being low-income (56%), food insecure (45%), and racial, cultural, or ethnic diversity (44%). Thirty-six percent of participants reported serving patient populations with substantial (≥20%) numbers of Hispanic patients. Fifty-eight percent of participants reported they promote or prescribe a specific diet to their patients and were asked to specify which diet or diets they prescribe. Of those, 48% prescribed a whole-food, plant-based diet, 39% prescribed a plant-predominant diet, 20% prescribed a Mediterranean diet, 16% prescribed a diet personalized to a specific patient/health condition, and 15% prescribed a whole food (minimally processed) diet.

**Table 1:** Respondent characteristics, practice, and patient population (*n* = 539).

	Mean (SD)
Age	49 (12)
	*N* (%)
Gender	
Female	415 (77.0)
Male	118 (21.9)
Non-binary/non-conforming	1 (0.2)
Prefer not to answer	5 (0.9)
Race	
White	397 (73.7)
Asian	69 (12.8)
Other	28 (5.2)
African American	25 (4.6)
American Indian or Alaska Native	5 (0.9)
Native Hawaiian or Other Pacific Islander	2 (0.4)
Prefer not to answer	20 (3.7)
Racial demographics within patient population (respondent estimated 20% or more of patients)	
White	435 (80.7)
Black or African-American	223 (41.4)
Asian	139 (25.8)
Prefer not to answer	50 (9.3)
American Indian or Alaska Native	24 (4.5)
Native Hawaiian or Other Pacific Islander	17 (3.2)
Ethnicity within patient population (respondent estimated 20% or more of patients Hispanic)	195 (36.2)
I prescribe or promote a specific diet to patients	314 (58.3)
(If yes) Diet prescribed/promoted	
Whole-food, plant-based	150 (47.8)
Plant-predominant	122 (38.9)
Mediterranean	62 (19.7)
Personalized to patient/health condition	51 (16.2)
Whole food/no or low processed food	46 (14.6)
Low carb/Keto	20 (6.4)
DASH Diet	18 (5.7)
Low fat	18 (5.7)
Self-described healthy diet/MyPlate	17 (5.4)
High/adequate protein	6 (1.9)
Other/not codable	10 (3.2)
Risk factors for health disparities faced by substantial numbers of patients (respondents estimated at least 20%)	
Low-income/underserved	302 (56.0)
Food insecurity/low access to healthy options	240 (44.5)
Racially, ethnically, and/or culturally diverse	237 (44.0)
Uninsured/underinsured	156 (28.9)
Low literacy	149 (27.6)
None of the above	123 (22.8)
Housing insecurity	119 (22.1)
Exposure to violence/trauma	116 (21.5)
Low English proficiency	115 (21.3)
Prefer not to answer	21 (3.9)
Do you assess diet with your patients?	
Yes, I discuss it during the visit informally	333 (61.8)
Yes, I do a formal assessment with a questionnaire or other tool	142 (26.4)
No, I would like to, but I face barriers	31 (5.8)
I am not a practitioner or am not practicing	24 (4.5)
No, it is not relevant in my practice	5 (0.9)
Prefer not to answer	4 (0.7)
With approximately what proportion of your patients do you discuss diet?	
All/most	393 (72.9)
Some	94 (17.4)
A few	25 (4.6)
None	15 (2.8)
Prefer not to answer	12 (2.2)
Do you face barriers when assessing patient’s diets?	
Yes, significant barriers	112 (20.8)
Yes, some barriers	320 (59.4)
No	80 (14.8)
Not applicable/prefer not to answer	27 (5.0)
What are the major barriers you face when assessing patients’ diets? (*n* = 528)	
Limited appointment time	239 (45.3)
Patient’s nutrition knowledge/understanding dietary habits	115 (21.8)
Patient’s ability/willingness to make changes	104 (19.7)
Recall/reporting bias	85 (16.1)
Lack of practitioner tools	65 (12.4)
Accuracy of assessment tools	57 (10.8)
Patient honesty about diet	55 (10.4)
Patient’s financial instability/cost of healthy food	34 (6.4)
Patient’s culture-familiarity w food-alignment of tool cuisine	30 (5.7)
Other^a^	144 (27.3)

^a^Includes wants more detail/more complete info/portion size/nutrients; unclear about portion size; compliance; relationship with patient; poor habits/lifestyle; response was not codable (did not answer question); lack of pay for assessment; not relevant to current practice; access to healthy foods; family/friends/social pressure; and patient lack of time.

The majority of participants reported assessing diet either informally (62%) or formally (26%) during patient encounters, and 73% reported discussing diet with their patients all or most of the time. Participants also reported facing some barriers (60%) or significant barriers (21%) to assessing diet. The top reported barrier was limited appointment time (45%).

Table 2 presents participant feedback on the screener. A majority of participants indicated they thought the screener captured the information they would want to know about patients’ diet very well (51%), somewhat well (30%), or extremely well (14%). Eighty-eight percent believed the screener was quick enough to use in a clinical setting, and 68% reported they would use it. Of these, participants reported they would use it most (46%), some (21%), or all (26%) of the time. Participants responded to the question asking if frequency without serving size was enough information with a “yes, frequency is enough information” (36%), “no, I would want to know about serving sizes” (31%), and “probably frequency alone is enough, but I’m not sure” (25%). After being presented with two examples, a bar chart and pie chart (Figure 1), of how to graphically present the summary scores, the majority of participants preferred the pie chart to the bar chart (70 vs. 12%). As far as potential missing items, 52% reported nothing they themselves ate was missing from the screener, 24% suggested additional foods should be added, and 8% suggested additional beverages should be added. When asked if there was anything else that would be important for them to know, a variety of topics were suggested, including other foods (15%), portion sizes (13%) eating behavior/preferences/allergies (12%), timing and/or frequency of eating (11%), and more detail about the food/diet (10%).

**Table 2:** Respondent feedback on ACLM Diet Screener (*n* = 539).

	*n* (%)
If you were to use this tool in clinical practice how well does it capture the information you would want to know about patients?	
Extremely well	77 (14.3)
Very well	272 (50.5)
Somewhat well	163 (30.2)
Not very well	17 (3.2)
Not well at all	6 (1.1)
Prefer not to answer	4 (0.7)
Respondents reporting that screener was quick enough to use in a clinical setting	473 (87.8)
Is frequency without serving size enough information?	
Yes, frequency is enough information	192 (35.6)
Probably frequency is enough, but I’m not sure	135 (25.1)
Probably frequency alone is NOT enough, but I’m not sure	44 (8.2)
No, I would want to know about serving sizes	166 (30.8)
Prefer not to answer	2 (0.4)
Would use the screener	368 (68.3)
(if yes) Approximately how often do you anticipate that you would use this screener in clinical practice? (*n* = 368)	
All of the time/with all my patients	97 (26.3)
Most of the time/with most of my patients	170 (46.2)
Some of the time/with some of my patients	77 (20.9)
Occasionally/with a few of my patients	14 (3.8)
Not applicable	7 (1.9)
Prefer not to answer	3 (1.0)
Do you prefer a bar chart or pie chart format for visualizing scoring and starting the conversation and/or educating patients?	
Pie chart	377 (69.9)
Bar chart	62 (11.5)
Both	68 (12.6)
Neither	23 (4.3)
Prefer not to answer	9 (1.7)
Is anything missing that you ate but that was not captured by the screener? (*n* = 402)^a^	
Nothing missing; satisfied with screener	210 (52.2)
Foods	98 (24.4)
Beverages	31 (7.7)
More detail about food and beverages	30 (7.5)
Supplements	28 (7.0)
Eating/cooking behavior/food prep	28 (7.0)
Food or beverage reported but already captured by screener	25 (6.2)
Not codable/did not answer question	14 (3.5)
Portion size	11 (2.7)
Is anything else missing that would be important for you to know as a practitioner but that was not captured by the screener? (*n* = 422)^a^	
Nothing missing; satisfied with screener	92 (21.8)
Food	62 (14.7)
Portion sizes	53 (12.6)
Eating behavior/preferences/allergies/	51 (12.1)
Timing and/or frequency of eating, including fasting	47 (11.1)
More detail about food/diet/energy intake/calories	42 (10.0)
Supplements	38 (9.0)
Food preparation	31 (7.3)
Non-codable/not answering question	22 (5.2)
Other^b^	115 (27.3)

^a^Free-text coded responses.

^b^Includes other health behaviors/risk factors; health literacy/nutrition knowledge; barriers/social determinants of health; beverages; scoring, interpreting, presenting data; water intake (amount/timing); tobacco/alcohol/drug consumption; different data collection method; patient goals/commitment/patient perception of health and eating.

The following key themes emerged from analysis of the final, free-text feedback question asking for any additional suggestions, which the team discussed and determined how to proceed: (1) feedback was positive, and participants seemed satisfied (no action); (2) some participants wanted more detail, portion sizes, or more questions were desired (study team felt these can be addressed in the planned long screener); (3) participants desired support in their practice that the screener cannot address, such as improving the doctor-patient relationship, patient honesty/recall bias, or patient economic circumstances (no action); (4) needs were identified that the screener already aims to address, such as brevity or the desire for a validated tool, or for questions that existed in the optional question list (continue as planned toward eventual validation); and (5) participants desired components that were already planned, such as translation (continue as planned toward translation, starting with Spanish). Overall, positive feedback was provided in regard to the value of the optional questions on food preferences, behaviors, self-efficacy and knowledge, and food insecurity (data not shown) (Table 3).

**Table 3:** Top^a^ feedback for changes to language, accuracy, efficacy, or clarity and action/planned action (*n* = 252).

Feedback	*n* (%)	Action/Planned action
No suggestions	128 (50.8)	N/A
More detail: define food categories, more examples	31 (12.3)	Added many more food examples; added images for clarity.
Translate to other languages	23 (9.1)	Plan to translate to Spanish first, then other languages if/when resources allow.
Add images of foods	16 (6.3)	Added images.
Revisit format of screener	13 (5.2)	Plan to offer paper version and eventually an electronic version; plan to translate to Spanish and eventually other languages; and added images and food examples.
Add food examples from more specific cultures/cuisines	10 (4.0)	Sought input from subject matter experts as well as reviewed other tools to incorporate a broader variety of foods in food examples.
Issues with health literacy/assess patient comprehension	8 (3.2)	Added additional food example details, as well as images.
Include portion sizes	8 (3.2)	Plan for long format questionnaire.

^a^Feedback reported by at least 3% of the sample.

The mean time to complete the screener was 3.4 min (SD = 2.4, range 1–20), using *n* = 518 responses with plausible values.

Following discussion, revision, and generation of multiple iterations of the screener, the final review of other diet assessment tools did result in the addition of a few items, not previously included. These were lard from the OPrevent FFQ (44), grits from the Block 2014 FFQ (45, 46), honey and molasses from the DSQ modified for use in the India Health Study (40), tamales and organ meat from the FFQ for the nutrition intervention for the Apache (43), mayonnaise from the FFQ developed for use in the southern United States (37), moose from the FFQ developed for Canadian First Nations (41), and chokeberries from the FFQ developed for the Navajo Nation (42).

The final version of the screener included 27 items, of which 19 were food categories, seven were beverage categories, and one was a nutrient or supplement (B12). Food categories included Fruit; Leafy green vegetables; Other vegetables, or vegetable dishes; Whole grains or whole grain products; Refined grains or refined grain products; Beans/legumes, or products made from them; Nuts, nut butters, seeds, avocado, or coconut; Meat or poultry or meat-based dishes; Fish or shellfish or seafood-based dishes; Eggs or egg-based dishes; Other dairy foods; Plant-based meat alternatives/mock meats; Dairy alternatives; Packaged/prepared foods or frozen meals; Restaurant/takeout foods; Fast foods; Packaged bars, shakes, or powders; Salty snacks or foods with added salt; Sweetened foods or foods w/added sugar; Fried foods or foods w/ added butter, fats, or oil. Beverage categories included Dairy milk; Non-dairy milk; Water or plain herbal beverages; 100% juice (fruit or vegetable); Beverages with added sugars/sweeteners; Coffee or other caffeinated beverages; and Alcoholic beverages. The final item was B12 supplement or B12-fortified foods. B12 was not included in the scoring.

The frequencies offered as answer choices were never, less than 1x/week, 1–3x/week, 4–6x/week, 1–2x/day, and more than 3x/day. Portion sizes were not assessed.

Summary scores for total whole plant food frequencies as a proportion of total food frequencies and total water frequency as a proportion of total beverage frequencies are quantitatively calculated as per the instructions displayed in Figure 1. For the purpose of clinical conversations with patients, and based on pilot testers’ preferences, we suggest displaying individual summary scores in a pie chart format and describing the scores as the proportion of whole plant foods out of total foods and proportion of water out of total beverages.

The ACLM Diet Screener Version 1, scoring instructions, and related materials can be accessed at https://lifestylemedicine.org/dietscreener.

## Discussion

This study details the iterative development of a brief diet screener that assesses intake of whole, plant foods in comparison to overall intake, provides summary scores to highlight the proportion of total plant food and total water consumption, and is appropriate for use by clinicians in brief patient encounters. The screener can be quickly reviewed by a physician, dietitian, or other member of a patient care team without quantitative analysis, simply by viewing the answers. Alternatively, if time and/or resources allow, a graphical summary of the output could be generated, as shown in Figure 1. A patient’s answers can be used to guide conversations around current diet, possible goals for behavior change, and to support tracking changes over time and use of nutrition prescriptions. This study followed a richly iterative process with robust pilot testing to gather feedback on the utility of the screener and possible improvements, laying a strong foundation for eventual validation.

To the authors’ knowledge, there are no existing dietary assessments that include all the characteristics of the screener developed here, specifically (1) capturing consumption of whole plant foods separately from other foods, (2) capturing the consumption of refined foods, foods with added sugar/salt/fats, or other unhealthy prepared/packaged foods, (3) capturing juice separately from fruit, (4) providing an answer to record zero consumption of a food item separately from infrequent or minimal, (5) made available freely to the public, and (6) generating summary scores capturing a dietary pattern of whole, plant foods in comparison to the overall diet. For these reasons, the ACLM Diet Screener is useful for clinicians writing nutrition prescriptions.

A number of tools exist for assessing diet, varying in length from single-question fruit and vegetable screeners to more comprehensive fruit and/or vegetable screeners (49) as well as questionnaires aimed at assessing fruit and vegetable intake along with overall diet (Block, Harvard semiquantitative FFQ) (36, 50). Of the 61 brief tools categorized as addressing fruit and vegetable intake on the RVSDAI at NCI (21), none adequately capture a picture of the overall diet, while simultaneously capturing total whole plant food intake and distinguishing between refined and unrefined foods. Additionally, none provide a simple summary measure that is clinically relevant by being both understandable and actionable for a patient. Supplementary Table 1 presents key characteristics of these tools. Among the RVSDAI tools, the number of question items ranged from 1 to 74, with the majority questionnaires having between 20 and 40 items.

In addition to assessment tools, there are also various scores and indices that can be applied to evaluate an individual’s diet, assuming the correct assessment was used to gather the data and provide the information needed for that evaluation. Some of these include the Healthy Eating Index (HEI) (51), Alternative Healthy Eating Index (AHEI) (29, 30), Mediterranean Diet Score (33), and the DASH Online Questionnaire (31, 32). The summary scores produced are often useful in research, and might be useful in clinical settings if data collection were possible. Each score focuses on different aspects of the diet, usually including both food and nutrient components. Our screener scoring is a simple calculation, enabling rapid feedback and straightforward interpretation by patients, without requiring nutrient calculations or complex statistical analysis. This is a strength of the screener, which makes it more accessible to users.

The ACLM Diet Screener fills the need for a clinical tool that LM practitioners can use to efficiently review dietary intake and, thus, begin a conversation around nutrition. Despite the knowledge that dietary intake significantly affects health, it is documented that practitioners have limited time to engage in a discussion of diet and lifestyle with their patients. For example, one recent study suggested that during a typical office day, physicians are only able to spend 27% of their time with patients directly, while nearly 50% of their time is allocated to administrative work including electronic health records (52). In the current study, 21% of respondents indicated significant barriers when it comes to assessing patient’s diets, and an additional 60% of respondents indicated some barriers. Nearly 50% of participants reported limited appointment time as a barrier to assessing patients’ diets. The ACLM Diet Screener is intended to be completed in less than 5 min and may be offered to patients to complete in the waiting room or before arriving at their visit. If scoring is applied, it may also serve as a tool to allow practitioners to review, within a matter of minutes, a snapshot of a patient’s dietary intake by viewing two summary scores representing consumption of (1) whole, plant foods and (2) water/plain herbal beverages. Importantly, 88% of respondents indicated that the screener was quick enough to use in a clinical setting.

While administering the screener electronically may be convenient for many patients and practitioners, a paper version is also available to be used in settings where resources do not allow for electronic data capture. In the long-term, we aim for the creation of a digital resource, such as an app, that would not only allow for electronic data capture via a smartphone but also produce a graphical summary of the data, such as presented in Figure 1, to further guide the practitioner in their conversation with a patient.

Sixty-eight percent of respondents indicated that they would use the screener, which highlights the utility of this tool. Furthermore, 95% reported that if they were to use the tool in clinical practice, it would capture the information that they would want to know (64% extremely or very well). With respect to the screener, over half of the participants reported that no items they ate were missed by the screener, and 22% reported that there was no other important information not captured by the screener.

The inclusion of food examples specific to African American, Hispanic/Latino, Asian American, Native American, and Indian American populations in the United States makes this screener unique. Currently, tools do exist for African-American, Hispanic/Latino, Asian-American, Native-American, and Indian-American populations; however, to the authors’ knowledge there is no single tool that attempts to generalize to all these populations, making our screener more flexible for a wider variety of patient populations.

While the screener is not intended to provide a comprehensive assessment of overall dietary intake, it focuses on whole, plant foods and water as these are two dietary components that are known to be lacking among individuals eating a standard American diet. In addition, it is well-established that plant-predominant diets, as well as diets low in processed foods, are effective for not only disease prevention, but also play a role in treatment of many chronic diseases, particularly cardiometabolic conditions (53). Adequate water intake is essential to health and noncommunicable disease prevention (54, 55). Clinical studies have clarified the benefits of water hydration and the avoidance of damage caused by fluid imbalance in both extracellular and intracellular water levels, particularly kidney dysfunction, kidney stones, poorer cognitive performance and brain function, and increased heart rate (56). Water’s importance for the prevention of nutrition-related noncommunicable diseases has emerged more recently because of the shift toward large proportions of fluids coming from caloric beverages (55). Calories taken in from beverages promotes central adiposity and increase risk for all cardiometabolic conditions, i.e., diabetes and heart disease (57–60). Average water intake in the United States is lower than recommended and is of particular concern in higher-risk groups such as Hispanic populations and older adults^.60^ Further, an estimated 20–22% of water intake comes from food, which is of particular concern in the United States where fruit and vegetable intake is known to be lower than recommended (55). The ACLM Diet Screener, uniquely aims to differentiate between not only whole plant-based foods vs. refined and non-plant-based foods, but also water vs. other beverages, which can help clinicians address the displacement of water with caloric drinks.

Strengths of this study included the richly iterative process used to develop the screener, the thorough review of SMEs with expertise in different cuisines, and the review of other previously validated tools. One limitation of this study is that the sample providing feedback were members of a professional organization for lifestyle medicine practitioners. While their feedback may not represent the general population of practitioners in theb United States, it does represent the sample of practitioners who have a high interest in food as medicine and a need for a screener assessing intake of unprocessed plant foods. It is also important to note that a relatively small proportion of invited participants completed the survey, yet this low response rate is not surprising for a research study in a population of busy healthcare professionals.

Another limitation of the screener itself is that foods are counted, when appropriate, in more than one category, such as foods that are refined and contain added sweeteners and fats. Thus, while the screener may be useful for initiating discussions around diet in a clinical setting, it may not accurately measure actual dietary intake. Similarly, while the line item for “water or plain herbal beverages” was created intentionally, with the goal of assessing unsweetened and non-caffeinated water intake, it may not accurately reflect total water intake or hydration status. Water, in particular, may be difficult for some people to quantify in terms of frequency if sipping continuously from a water bottle. The ability of the screener to quantify both food and beverage intake will be further assessed in a future validation study. In addition, the calculation of the summary score for beverages, which does group beverages contributing toward nutrient requirements (dairy milk, plant-based milk, and juice) with beverages that provide empty calories (sugar-sweetened beverages and alcohol). However, as discussed, water consumption is frequently inadequate in the United States, and increasing water consumption is often a goal in lifestyle medicine practice. While some consumption of energy-containing fluid may positively contribute toward nutrient requirements, water should be the beverage of choice, as noted in the Dietary Guidelines for Americans (61) and Canadian Dietary Guidelines (62). The summary score can be used to draw attention to overall fluid consumption for goal setting.

Future research will first involve conducting a pilot study to utilize the screener in a small number of clinical practices. Such a study will assess use of the screener in a real-life, clinical setting, as well as including usefulness of the scoring. A formal validation study will be conducted to validate the screener against multi-day food records, first in the general United States population and later in specific ethnic and cultural groups to ensure relevance.

## Conclusion

The ACLM Diet Screener was developed through iterative review and pilot testing. The screener is a brief, 27-item diet assessment tool that can be successfully used in clinical settings to track patient dietary intakes, guide clinical conversations, and support nutrition prescriptions. Pilot testing of the screener found strong alignment with clinician needs for assessing overall dietary patterns of patients. Future research will involve pilot testing the screener in clinical interventions and conducting a validation study to establish construct validity.

## Data availability statement

The datasets presented in this article are not readily available because individuals may request access to the data by contacting the corresponding author. Requests to access the datasets should be directed to mkarlsen@lifestylemedicine.org.

## Ethics statement

This study involving humans was approved by University of New England Institutional Review Board. The study was conducted in accordance with the local legislation and institutional requirements. The ethics committee/institutional review board waived the requirement of written informed consent for participation from the participants or the participants’ legal guardians/next of kin because this was deemed a low-risk study. Participants were asked to review a participant information sheet with details of the study, and consent was implied by starting the survey. Written informed consent was not required by the IRB.

## Author contributions

MK: Project administration, Writing – original draft, Writing – review & editing, Conceptualization, Formal analysis, Investigation, Methodology, Supervision. KS: Project administration, Writing – original draft, Writing – review & editing, Data curation. KP: Data curation, Writing – review & editing. KC: Writing – review & editing. SH: Data curation, Writing – review & editing. EC: Writing – review & editing. SF: Writing – review & editing.
