# Supplementary material for: Piloting a brief assessment to capture consumption of whole plant food and water: version 1.0 of the American College of Lifestyle Medicine Diet Screener (ACLM Diet Screener)
Source: Front Nutr. 2024 Apr 26;11:1356676. doi: 10.3389/fnut.2024.1356676 (PMC11085256; doi:10.3389/fnut.2024.1356676)
Supplement: Supplementary file 1 [file Table_1.DOCX]

**Supplemental Table: Summary of Short Dietary Assessment Instruments Capturing Fruit and Vegetable Intake^a^**

| **Validation Study** | **Name of Instrument, if applicable** | **Dietary Factors Assessed** | **Format** | **Number**  **of**  **Items** | **Race of Validation Study Population** |
| --- | --- | --- | --- | --- | --- |
| Alcantara I^1^ | BRFSS Fruit and Vegetable Consumption Module | fruits and vegetables | FFQ | 6 | African-American |
| Andersen LF^2^ |  | fruits and vegetables, fat | FFQ | 27 | Not specified |
| Apovian CM^3^ | DASH Online Questionnaire | fruits and vegetables, whole grains, low-fat dairy products, meats, sugary sweets, fat, nuts/seeds/legumes | checklist | 74 | Not specified |
| Ashton L^4^ | The Fruit And Vegetable Variety index (FAVVA) | fruits and vegetables | FFQ | 35 | Not specified |
| Béliard S^5^ | NLSChol Questionnaire | fruits and vegetables, dietary fats, bread and carbohydrates, phytosterols | FFQ | 11 | Not specified |
| Bell LK^6^ | Toddler Dietary Questionnaire (TDQ) | fruits and vegetables, dairy, high-fat foods, high-sugar foods, high-salt foods, sweetened beverages | FFQ | 19 | Not specified |
| Bleiweiss-Sande R^7^ | Fueling Learning through Exercise (FLEX) dietary questionnaire | fruits and vegetables, snacks, beverages | FFQ | 39 | Not specified |
| Block G^8^ |  | fruits and vegetables, fats | FFQ | 22 | Multiple |
| Bogers RP^9^ |  | fruits and vegetables | FFQ | 8 | Not specified |
| Bully P^10^ | Prescribe Healthy Life Screening Questionnaire (Prescribe Vida Saludable; PVS-SQ) | fruits and vegetables | FFQ | 12 (2 specifically on diet) | Not specified |
| Cook A^11^ | Five-item questionnaire for vegetable | vegetables | FFQ | 5 | Not specified |
| Cook A^11^ | Single Question (SQ) for Fruit | fruits | FFQ | 1 | Not specified |
| Cook A^11^ | Single Question (SQ) for Vegetable | vegetables | FFQ | 1 | Not specified |
| Cullen KW^12^ | Fruit, juice and vegetable (FJV) FFQ | fruits and vegetables (fruit, juice, and vegetables) | FFQ | 24 | Mostly Black |
| Eaton DK^13^ | CDC's Youth Risk Behavior Surveillance System (YRBSS) Fruit and Vegetable Intake Questions and Alternative Questions | fruits and vegetables | FFQ | 6; 6; 2 | Not specified |
| Field AE^14^ | Behavioral Risk Factor Surveillance System (BRFSS) questionaire | fruits and vegetables | FFQ | 6 | Multiple |
| Field AE^14^ | Youth Risk Behavior Surveillance System (YRBSS) questionnaire | fruits and vegetables | FFQ | 4 | Multiple |
| Flood VM^15^ |  | fruits and vegetables, lean meat, processed meat, take-away food, snack foods, potato crisps and confectionary, soft drinks/cordials, juice, milk, water | FFQ | 17 | Not specified |
| Fowke JH^16^ |  | cruciferous vegetables | FFQ | 37 | Not specified |
| Gadowski AM^17^ | Australian Short Dietary Screener (Aus-SDS) | vegetables, fruits, legumes and beans, cereals, protein sources and dairy sources | FFQ | 6 | Not specified |
| Gans KM^18^ | Rapid Eating and Activity Assessment (REAP) | fruits and vegetables, whole grains, calcium-rich foods, fat, saturated fat and cholesterol, sugar-containing foods and beverages, sodium, alcoholic beverages | FFQ | 27 | Mostly white |
| Gilsing A^19^ | Short Diet Questionnaire (SDQ) | fruits and vegetables, fats, fiber, calcium, vitamin D, whole grains, calcium-fortified foods and beverages | FFQ | 30 | Not specified |
| Greene GW^20^ | NCI fruits and vegetables screener | fruits and vegetables | FFQ | 19 | Multiple |
| Hendrie GA^21^ |  | fruits and vegetables, milk type | FFQ | 3 | Not specified |
| Hendrie GA^22^ | Short Food Survey | fruits and vegetables, breads and cereals, meat and alternatives, dairy, beverages | FFQ | 38 | Not specified |
| Huang YC^23^ | Simplified food frequency questionnaire | fruits and vegetables, food group patterns, grain, dairy, meat, fish, egg, soy | FFQ | 37 | Fukienese, Hakka, Mainlander, Aboriginal |
| Hunsberger M^24^ | Block Kids Food Screener | fruits and vegetables, dairy, whole grains, protein sources, saturated fat, added sugar | FFQ | 41 | Not specified |
| Jilcott SB^25^ | Modified dietary risk assessment | fruits and vegetables, fat, fiber | Index | 54 | Not specified |
| Johnson F^26^ | Adolescent Food Habits Checklist | fruits and vegetables, healthy eating behaviors, energy-dense foods | behavioral questionnaire | 23 | Mostly white |
| Kristal AR^27^ | 5-A-DAY questionnaire | fruits and vegetables | FFQ | 7 | Multiple |
| Laviolle B^28^ |  | fruits and vegetables, saturated fatty acids, mono-unsaturated fatty acids, polyunsaturated fatty acids | FFQ | 14 | Not specified |
| Lean MEJ^29^ | Dietary Targets Monitor | fruits and vegetables, fish, starchy foods | FFQ | 9 | Not specified |
| Leppälä J^30^ | Index of Diet Quality | fruits, vegetables, and berries; whole grain foods; fat-containing foods; sugar-rich foods; dairy; supplements; meal pattern; alcohol | FFQ | 55 | Not specified |
| Lillegaard ITL^31^ |  | fruits and vegetables, high-fat foods, high-sugar foods | FFQ | 23 | Not specified |
| Ling AM^32^ |  | fruits and vegetables, cereals | FFQ | 16 | Not specified |
| Magarey A^33^ | Children's Dietary Questionnaire | fruits and vegetables, fat from dairy, sweetened beverages, non-core foods | FFQ | 28 | Not specified |
| Mainvil LA^34^ |  | fruits and vegetables | FFQ | 5-item and 12-item (2 fruit questionnaires); 5-item and 15-item (2 vegetable questionnaires) | Largely European |
| Murphy S^35^ | Food Behavior Checklist | fruits and vegetables, fat, fiber, dairy, salt, diety quality, food security, food expenditure | Checklist | 39 | Multiple |
| Neuhouser^36^ | Beverage and snack questionnaire | fruits and vegetables, soft drinks, salty snacks, sweets, milk | FFQ | 19 | Multiple |
| Plaete J^37^ | Fruit Test | fruits | FFQ | 17 | Not specified |
| Plaete J^37^ | Vegetable Test | vegetables | FFQ | 13 | Not specified |
| Prochaska JJ^38^ | Fruit and vegetable brief | fruits and vegetables | FFQ | 2 | Multiple |
| Resincow K^39^ |  | fruits and vegetables | FFQ | 2 | Black |
| Resincow K^39^ |  | fruits and vegetables | FFQ | 7 | Black |
| Resincow K^39^ |  | fruits and vegetables | FFQ | 36 | Black |
| Rifas-Shiman^40^ | PrimeScreen | fruits and vegetables, whole grains, dairy, fish, red meats, fatty foods, vitamins and supplements | FFQ | 25 | Multiple |
| Serdula M^41^ | BRFSS fruits and vegetable module | fruits and vegetables | FFQ | 6 | Multiple |
| Spencer EH^42^ | Healthy doc diet screener | fruits and vegetables, fat | FFQ | 43 | Multiple |
| Thompson FE (Colón-Ramos et al)^43^ | CHIS 2005 2-factor screener (NCI) | fruits and vegetables, added sugars | FFQ | 11 | Hispanic |
| Thompson FE^44^ | NCI All-day screener | fruits and vegetables | FFQ | 9 | Not specified |
| Thompson FE^44^ | NCI By-meal screener | fruits and vegetables | FFQ | 13, 14 | Not specified |
| Thompson FE^45^ | NCI Multifactor screener | fruits and vegetables, energy from fat and fiber | FFQ | 17 | Not specified |
| Thompson FE^46^ | NCI new F&V screener | fruits and vegetables | FFQ | 16 | Multiple |
| Thompson FE^46^ | NCI standard F&V screener | fruits and vegetables | FFQ | 7 | Multiple |
| Thompson FE^47^ | NHIS 5-factor screener (NCI) | fruits and vegetables, added sugar, fiber, calcium and dairy servings | FFQ | 20 | Multiple |
| Townsend MS^48^ | Focus on Veggies | fruits and vegetables |  | 10 | Multiple |
| Yaroch AL^49^ | 2-item cup fruit and vegetable screener | fruits and vegetables | FFQ | 2 | Black, white |
| Yaroch AL^49^ | 2-item serving fruit and vegetable screener | fruits and vegetables | FFQ | 2 | Black, white |
| Yaroch AL^49^ | 8-item fruit and vegetable screener | fruits and vegetables | FFQ | 8 | Black, white |
| Zhang Y^50^ | Food Web Questionnaire (FWQ) | fruits and vegetables | behavioral questionnaire | 8 | Multiple |
| Zhang Y^50^ | Plate Activity Questionnaire (PAQ) | fruits and vegetables | behavioral questionnaire | 1 | Multiple |

**^a^** identified using the NCI Register of Validated Short Dietary Assessment Instruments

**References**

1. Alcantara I, Haardorfer R, Gazmararian JA, Hartman TJ, Greene B, Kegler MC. Relative validation of fruit and vegetable intake and fat intake among overweight and obese African-American women. *Public Health Nutr*. Aug 2015;18(11):1932-40. doi:10.1017/S1368980014002547

2. Andersen LF, Johansson L, Solvoll K. Usefulness of a short food frequency questionnaire for screening of low intake of fruit and vegetable and for intake of fat. *Eur J Public Health*. Sep 2002;12(3):208-13. doi:10.1093/eurpub/12.3.208

3. Apovian CM, Murphy MC, Cullum-Dugan D, et al. Validation of a web-based dietary questionnaire designed for the DASH (dietary approaches to stop hypertension) diet: the DASH online questionnaire. *Public Health Nutr*. May 2010;13(5):615-22. doi:10.1017/S1368980009991996

4. Ashton L, Williams R, Wood L, et al. The comparative validity of a brief diet screening tool for adults: The Fruit And Vegetable VAriety index (FAVVA). *Clin Nutr ESPEN*. Feb 2019;29:189-197. doi:10.1016/j.clnesp.2018.10.007

5. Beliard S, Coudert M, Valero R, et al. Validation of a short food frequency questionnaire to evaluate nutritional lifestyles in hypercholesterolemic patients. *Ann Endocrinol (Paris)*. Dec 2012;73(6):523-9. doi:10.1016/j.ando.2012.09.006

6. Bell LK, Golley RK, Magarey AM. A short food-group-based dietary questionnaire is reliable and valid for assessing toddlers' dietary risk in relatively advantaged samples. *Br J Nutr*. Aug 28 2014;112(4):627-37. doi:10.1017/S0007114514001184

7. Bleiweiss-Sande R, Kranz S, Bakun P, Tanskey L, Wright C, Sacheck J. Comparative Study of a New Dietary Screener to Assess Food Groups of Concern in Children. *Food Nutr Bull*. Dec 2017;38(4):585-593. doi:10.1177/0379572117733400

8. Block G, Gillespie C, Rosenbaum EH, Jenson C. A rapid food screener to assess fat and fruit and vegetable intake. *Am J Prev Med*. May 2000;18(4):284-8. doi:10.1016/s0749-3797(00)00119-7

9. Bogers RP, Van Assema P, Kester AD, Westerterp KR, Dagnelie PC. Reproducibility, validity, and responsiveness to change of a short questionnaire for measuring fruit and vegetable intake. *Am J Epidemiol*. May 1 2004;159(9):900-9. doi:10.1093/aje/kwh123

10. Bully P, Sanchez A, Grandes G, et al. Metric properties of the "prescribe healthy life" screening questionnaire to detect healthy behaviors: a cross-sectional pilot study. *BMC Public Health*. Dec 7 2016;16(1):1228. doi:10.1186/s12889-016-3898-8

11. Cook A, Roberts K, O'Leary F, Allman-Farinelli MA. Comparison of single questions and brief questionnaire with longer validated food frequency questionnaire to assess adequate fruit and vegetable intake. *Nutrition*. Jul-Aug 2015;31(7-8):941-7. doi:10.1016/j.nut.2015.01.006

12. Cullen KW, Baranowski T, Baranowski J, Hebert D, de Moor C. Pilot study of the validity and reliability of brief fruit, juice and vegetable screeners among inner city African-American boys and 17 to 20 year old adults. *J Am Coll Nutr*. Oct 1999;18(5):442-50. doi:10.1080/07315724.1999.10718882

13. Eaton DK, Olsen EO, Brener ND, et al. A comparison of fruit and vegetable intake estimates from three survey question sets to estimates from 24-hour dietary recall interviews. *Journal of the Academy of Nutrition and Dietetics*. Sep 2013;113(9):1165-74. doi:10.1016/j.jand.2013.05.013

14. Field AE, Colditz GA, Fox MK, et al. Comparison of 4 questionnaires for assessment of fruit and vegetable intake. *Am J Public Health*. Aug 1998;88(8):1216-8. doi:10.2105/ajph.88.8.1216

15. Flood VM, Wen LM, Hardy LL, Rissel C, Simpson JM, Baur LA. Reliability and validity of a short FFQ for assessing the dietary habits of 2-5-year-old children, Sydney, Australia. *Public Health Nutr*. Mar 2014;17(3):498-509. doi:10.1017/S1368980013000414

16. Fowke JH, Hebert JR, Fahey JW. Urinary excretion of dithiocarbamates and self-reported Cruciferous vegetable intake: application of the 'method of triads' to a food-specific biomarker. *Public Health Nutr*. Dec 2002;5(6):791-9. doi:10.1079/PHN2002345

17. Gadowski AM, McCaffrey TA, Heritier S, et al. Development, Relative Validity and Reproducibility of the Aus-SDS (Australian Short Dietary Screener) in Adults Aged 70 Years and Above. *Nutrients*. May 15 2020;12(5)doi:10.3390/nu12051436

18. Gans KM, Risica PM, Wylie-Rosett J, et al. Development and evaluation of the nutrition component of the Rapid Eating and Activity Assessment for Patients (REAP): a new tool for primary care providers. *J Nutr Educ Behav*. Sep-Oct 2006;38(5):286-92. doi:10.1016/j.jneb.2005.12.002

19. Gilsing A, Mayhew AJ, Payette H, et al. Validity and Reliability of a Short Diet Questionnaire to Estimate Dietary Intake in Older Adults in a Subsample of the Canadian Longitudinal Study on Aging. *Nutrients*. Oct 17 2018;10(10)doi:10.3390/nu10101522

20. Greene GW, Resnicow K, Thompson FE, et al. Correspondence of the NCI Fruit and Vegetable Screener to repeat 24-H recalls and serum carotenoids in behavioral intervention trials. *J Nutr*. Jan 2008;138(1):200S-204S. doi:10.1093/jn/138.1.200S

21. Hendrie GA, Riley MD. Performance of short food questions to assess aspects of the dietary intake of Australian children. *Nutrients*. Nov 26 2013;5(12):4822-35. doi:10.3390/nu5124822

22. Hendrie GA, Viner Smith E, Golley RK. The reliability and relative validity of a diet index score for 4-11-year-old children derived from a parent-reported short food survey. *Public Health Nutr*. Jul 2014;17(7):1486-97. doi:10.1017/S1368980013001778

23. Huang Y, Lee M, Pan W, Wahlqvist ML. Validation of a simplified food frequency questionnaire as used in the Nutrition and Health Survey in Taiwan (NAHSIT) for the elderly. *Asia Pac J Clin Nutr*. 2011;20(1):134-40.

24. Hunsberger M, O'Malley J, Block T, Norris JC. Relative validation of Block Kids Food Screener for dietary assessment in children and adolescents. *Matern Child Nutr*. Apr 2015;11(2):260-70. doi:10.1111/j.1740-8709.2012.00446.x

25. Jilcott SB, Keyserling TC, Samuel-Hodge CD, Johnston LF, Gross MD, Ammerman AS. Validation of a brief dietary assessment to guide counseling for cardiovascular disease risk reduction in an underserved population. *J Am Diet Assoc*. Feb 2007;107(2):246-55. doi:10.1016/j.jada.2006.11.006

26. Johnson F, Wardle J, Griffith J. The Adolescent Food Habits Checklist: reliability and validity of a measure of healthy eating behaviour in adolescents. *European journal of clinical nutrition*. Jul 2002;56(7):644-9. doi:10.1038/sj.ejcn.1601371

27. Kristal AR, Vizenor NC, Patterson RE, Neuhouser ML, Shattuck AL, McLerran D. Precision and bias of food frequency-based measures of fruit and vegetable intakes. *Cancer Epidemiol Biomarkers Prev*. Sep 2000;9(9):939-44.

28. Laviolle B, Froger-Bompas C, Guillo P, et al. Relative validity and reproducibility of a 14-item semi-quantitative food frequency questionnaire for cardiovascular prevention. *Eur J Cardiovasc Prev Rehabil*. Dec 2005;12(6):587-95. doi:10.1097/01.hjr.0000186625.98604.5d

29. Lean ME, Anderson AS, Morrison C, Currall J. Evaluation of a dietary targets monitor. *European journal of clinical nutrition*. May 2003;57(5):667-73. doi:10.1038/sj.ejcn.1601596

30. Leppala J, Lagstrom H, Kaljonen A, Laitinen K. Construction and evaluation of a self-contained index for assessment of diet quality. *Scand J Public Health*. Dec 2010;38(8):794-802. doi:10.1177/1403494810382476

31. Lillegaard IT, Overby NC, Andersen LF. Evaluation of a short food frequency questionnaire used among Norwegian children. *Food Nutr Res*. 2012;56doi:10.3402/fnr.v56i0.6399

32. Ling AM, Horwath C, Parnell W. Validation of a short food frequency questionnaire to assess consumption of cereal foods, fruit and vegetables in Chinese Singaporeans. *European journal of clinical nutrition*. Aug 1998;52(8):557-64. doi:10.1038/sj.ejcn.1600605

33. Magarey A, Golley R, Spurrier N, Goodwin E, Ong F. Reliability and validity of the Children's Dietary Questionnaire; a new tool to measure children's dietary patterns. *Int J Pediatr Obes*. 2009;4(4):257-65. doi:10.3109/17477160902846161

34. Mainvil LA, Horwath CC, McKenzie JE, Lawson R. Validation of brief instruments to measure adult fruit and vegetable consumption. *Appetite*. Feb 2011;56(1):111-7. doi:10.1016/j.appet.2010.09.008

35. Murphy SP, Kaiser LL, Townsend MS, Allen LH. Evaluation of validity of items for a food behavior checklist. *J Am Diet Assoc*. Jul 2001;101(7):751-61. doi:10.1016/S0002-8223(01)00189-4

36. Neuhouser ML, Lilley S, Lund A, Johnson DB. Development and validation of a beverage and snack questionnaire for use in evaluation of school nutrition policies. *J Am Diet Assoc*. Sep 2009;109(9):1587-92. doi:10.1016/j.jada.2009.06.365

37. Plaete J, De Bourdeaudhuij I, Crombez G, et al. The Reliability and Validity of Short Online Questionnaires to Measure Fruit and Vegetable Intake in Adults: The Fruit Test and Vegetable Test. *PLoS One*. 2016;11(7):e0159834. doi:10.1371/journal.pone.0159834

38. Prochaska JJ, Sallis JF. Reliability and validity of a fruit and vegetable screening measure for adolescents. *J Adolesc Health*. Mar 2004;34(3):163-5. doi:10.1016/j.jadohealth.2003.07.001

39. Resnicow K, Odom E, Wang T, et al. Validation of three food frequency questionnaires and 24-hour recalls with serum carotenoid levels in a sample of African-American adults. *Am J Epidemiol*. Dec 1 2000;152(11):1072-80. doi:10.1093/aje/152.11.1072

40. Rifas-Shiman SL, Willett WC, Lobb R, Kotch J, Dart C, Gillman MW. PrimeScreen, a brief dietary screening tool: reproducibility and comparability with both a longer food frequency questionnaire and biomarkers. *Public Health Nutr*. Apr 2001;4(2):249-54. doi:10.1079/phn200061

41. Serdula M, Coates R, Byers T, et al. Evaluation of a brief telephone questionnaire to estimate fruit and vegetable consumption in diverse study populations. *Epidemiology*. Sep 1993;4(5):455-63. doi:10.1097/00001648-199309000-00012

42. Spencer EH, Elon LK, Hertzberg VS, Stein AD, Frank E. Validation of a brief diet survey instrument among medical students. *J Am Diet Assoc*. May 2005;105(5):802-6. doi:10.1016/j.jada.2005.02.003

43. Colon-Ramos U, Thompson FE, Yaroch AL, et al. Differences in fruit and vegetable intake among Hispanic subgroups in California: results from the 2005 California Health Interview Survey. *J Am Diet Assoc*. Nov 2009;109(11):1878-85. doi:10.1016/j.jada.2009.08.015

44. Thompson FE, Subar AF, Smith AF, et al. Fruit and vegetable assessment: performance of 2 new short instruments and a food frequency questionnaire. *J Am Diet Assoc*. Dec 2002;102(12):1764-72. doi:10.1016/s0002-8223(02)90379-2

45. Thompson FE, Midthune D, Subar AF, Kahle LL, Schatzkin A, Kipnis V. Performance of a short tool to assess dietary intakes of fruits and vegetables, percentage energy from fat and fibre. *Public Health Nutr*. Dec 2004;7(8):1097-105. doi:10.1079/PHN2004642

46. Thompson FE, Kipnis V, Subar AF, et al. Evaluation of 2 brief instruments and a food-frequency questionnaire to estimate daily number of servings of fruit and vegetables. *Am J Clin Nutr*. Jun 2000;71(6):1503-10. doi:10.1093/ajcn/71.6.1503

47. Thompson FE, McNeel TS, Dowling EC, Midthune D, Morrissette M, Zeruto CA. Interrelationships of added sugars intake, socioeconomic status, and race/ethnicity in adults in the United States: National Health Interview Survey, 2005. *J Am Diet Assoc*. Aug 2009;109(8):1376-83. doi:10.1016/j.jada.2009.05.002

48. Townsend MS, Shilts MK, Styne DM, et al. Vegetable behavioral tool demonstrates validity with MyPlate vegetable cups and carotenoid and inflammatory biomarkers. *Appetite*. Dec 1 2016;107:628-638. doi:10.1016/j.appet.2016.09.002

49. Yaroch AL, Tooze J, Thompson FE, et al. Evaluation of three short dietary instruments to assess fruit and vegetable intake: the National Cancer Institute's food attitudes and behaviors survey. *Journal of the Academy of Nutrition and Dietetics*. Oct 2012;112(10):1570-7. doi:10.1016/j.jand.2012.06.002

50. Zhang Y, Reicks M. Test-Retest Reliability and Convergent Validity of Two Brief Fruit and Vegetable Intake Questionnaires among School-Aged Children. *Nutrients*. Jul 6 2017;9(7)doi:10.3390/nu9070707
